# Involvement of KCa3.1 channel activity in immediate perioperative cognitive and neuroinflammatory outcomes

**DOI:** 10.1186/s12871-023-02030-2

**Published:** 2023-03-16

**Authors:** Sarah Saxena, Vincent Nuyens, Christopher Rodts, Kristina Jamar, Adelin Albert, Laurence Seidel, Mustapha Cherkaoui-Malki, Jean G. Boogaerts, Heike Wulff, Mervyn Maze, Véronique Kruys, Joseph Vamecq

**Affiliations:** 1 Department of Anesthesia and Critical Care, AZ Sint-Jan Brugge Oostende AV, Bruges, Belgium; 2 Experimental Medicine Laboratory, ULB 222 Unit, CHU-Charleroi, Université Libre de Bruxelles, Montigny-Le-Tilleul, Belgium; 3 Department of Anesthesiology, CHU-Charleroi, Université Libre de Bruxelles, Charleroi, Belgium; 4 Department of Biostatistics (B-STAT), University Hospital of Liège, Liège, Belgium; 5 Laboratoire Bio-PeroxIL EA7270, Univ. Bourgogne Franche-Comté, 6 Bd Gabriel, 21000 Dijon, France; 6 Department of Pharmacology, University of California Davis, Davis, CA USA; 7 Center for Cerebrovascular Research, Department of Anesthesia and Perioperative Care, UCSF, San Francisco, CA USA; 8 Laboratory of Molecular Biology of the Gene, Department of Molecular Biology, Université Libre de Bruxelles (ULB), Gosselies, Belgium; 9 Inserm, Biochemistry and Molecular Biology Laboratory, HMNO, CBP, CHU Lille & EA 7364 - RADEME, North France University Lille, Lille, France

**Keywords:** Neuroinflammation, Surgery, Post-operative cognitive decline, Perioperative neurocognitive disorders, Inflammation, Cognition, Anesthesia, Microglia

## Abstract

**Background:**

Potassium channels (KCa3.1; Kv1.3; Kir2.1) are necessary for microglial activation, a pivotal requirement for the development of Perioperative Neurocognitive Disorders (PNDs). We previously reported on the role of microglial Kv1.3 for PNDs; the present study sought to determine whether inhibiting KCa3.1 channel activity affects neuroinflammation and prevents development of PND.

**Methods:**

Mice (*wild-type* [*WT*] and *KCa3.1*^*−/−*^) underwent aseptic tibial fracture trauma under isoflurane anesthesia or received anesthesia alone. *WT* mice received either TRAM34 (a specific KCa3.1 channel inhibitor) dissolved in its vehicle (miglyol) or miglyol alone. Spatial memory was assessed in the Y-maze paradigm 6 h post-surgery/anesthesia. Circulating interleukin-6 (IL-6) and high mobility group box-1 protein (HMGB1) were assessed by ELISA, and microglial activitation Iba-1 staining.

**Results:**

In *WT* mice surgery induced significant cognitive decline in the Y-maze test, p = 0.019), microgliosis (p = 0.001), and increases in plasma IL-6 (p = 0.002) and HMGB1 (p = 0.001) when compared to anesthesia alone. TRAM34 administration attenuated the surgery-induced changes in cognition, microglial activation, and HMGB1 but not circulating IL-6 levels. In *KCa3.1*^*−/−*^ mice surgery neither affected cognition nor microgliosis, although circulating IL-6 levels did increase (p < 0.001).

**Conclusion:**

Similar to our earlier report with Kv1.3, perioperative microglial KCa3.1 blockade decreases immediate perioperative cognitive changes, microgliosis as well as the peripheral trauma marker HMGB1 although surgery-induced IL-6 elevation was unchanged. Future research should address whether a synergistic interaction exists between blockade of Kv1.3 and KCa3.1 for preventing PNDs.

**Supplementary Information:**

The online version contains supplementary material available at 10.1186/s12871-023-02030-2.

## Background

Perioperative neurocognitive disorder (PND), first described in 1887, [1] is a frequent misdiagnosed complication [2–4]. While several etiological factors have been investigated [5–7], the most plausible explanation implicates trauma-induced inflammation for the development of PND [8–10].

During surgical incision, traumatized tissues release high mobility group box protein1 (HMGB1) into the bloodstream [8]. This damage-associated molecular pattern binds to pattern recognition receptors on circulating, CCR2^+^ bone marrow-derived monocytes (BM-DMs), triggering the nuclear translocation of the transcription factor NF-kB which activates gene expression and release of pro-inflammatory cytokines including IL-6 and IL-1β [8] and leads to blood-brain barrier disruption [9]. Within the brain parenchyma, the chemokine MCP-1 (also referred to as CCL2) is upregulated and, by signaling through its receptor, attracts CCR2^+^ BM-DMs [10]; the resulting influx of BM-DMs into brain activates quiescent microglia. Together, BM-DMs and activated microglia release HMGB1, IL-6, and IL-1β, thereby disrupting long-term potentiation and the synaptic plasticity involved in the cognitive functions of learning and memory [11–13]. Exaggerated or unresolved inflammation promotes the development of PND [4, 14–16].

Among strategies that have been investigated to mitigate the development of PND, preventing the activation of microglia may be one of the most promising. Microglia surveil the milieu and are activated by changes in its environment. When differentiated into the pro-inflammatory (“M1”) phenotype, the activated microglia synthesize and release cytokines that propagate the inflammatory process; conversely, when transformed into the anti-inflammatory phenotype (“M2”) microglia support regeneration. M1 microglia play an active role in the development of PNDs following peripheral surgery [10].

Microglia express multiple potassium (K^+^) channels (KCa3.1; Kv1.3; Kir2.1), which are up-regulated during activation and participate in microglial calcium signaling and neuroinflammation [17]. We previously reported that Kv1.3 channel blockade mitigated PND development [18]. KCa3.1 is a voltage-independent, homo-tetrameric potassium channel which is constitutively associated with its calcium sensor calmodulin and therefore opens in response to increased intracellular calcium (above ~ 150 nM). In turn, a negative membrane potential is maintained through K^+^ efflux [17, 19]. The channel is expressed on proliferating fibroblasts, on dedifferentiated vascular smooth muscle cells, and on immune cells including microglia and macrophages, activated CCR7^+^ T cells and IgD^+^ B cells [17, 19].

In microglia, KCa3.1 has been shown to be involved in respiratory bursting, migration, proliferation, and nitric oxide production, as well neuroapoptosis in organotypic hippocampal slices, suggesting that KCa3.1 suppression may be useful for in neurological diseases featuring microglial activation [19–21]. TRAM34, a small molecule inhibitor, blocks the KCa3.1 channel with an IC_50_ of 20 nmol/L and exhibits 200- to 1,500-fold selectivity over other K^+^ channels [19].

The present study investigated whether blockade of microglial KCa3.1 channel activity impacts the trauma-induced inflammatory cascade and protect against the development of PND.

## Methods

### Animal care

Experimental procedures involving animals were approved by the Animal Care Committee of the Université Libre de Bruxelles. (CEBEA-IBMM 2019-24-105).

Twelve weeks old C57BL/6J mice (Charles River Laboratories, France) and *KCa3.1*^*−/−*^ mice (genetic background: C57BL/6J; Mouse Biology Program UC Davis) [20] were separately group-housed with 12 h light/dark cycles in a temperature-controlled environment with *ad libitum* access to standard rodent chow and water in our animal research facilities during one week prior to experiments.

### Experimental groups

12-week-old wild type mice were randomly assigned to surgery (anesthesia followed by surgery) or anesthesia alone groups that either received vehicle or TRAM34 (T) treatment:

Group 1: Anesthesia (+ vehicle).

Group 2: Surgery (+ vehicle).

Group 3: Anesthesia + T(TRAM34).

Group 4: Surgery + T(TRAM34).

12 weeks-old KCa3.1^−/−^ mice were randomized to surgery (anesthesia followed by surgery) or anesthesia alone groups:

Group 1: Anesthesia (without vehicle or TRAM34).

Group 2: Surgery (without vehicle or TRAM34).

In all experiments, 8 to 10 mice were included per group.

**Fig. 1:**
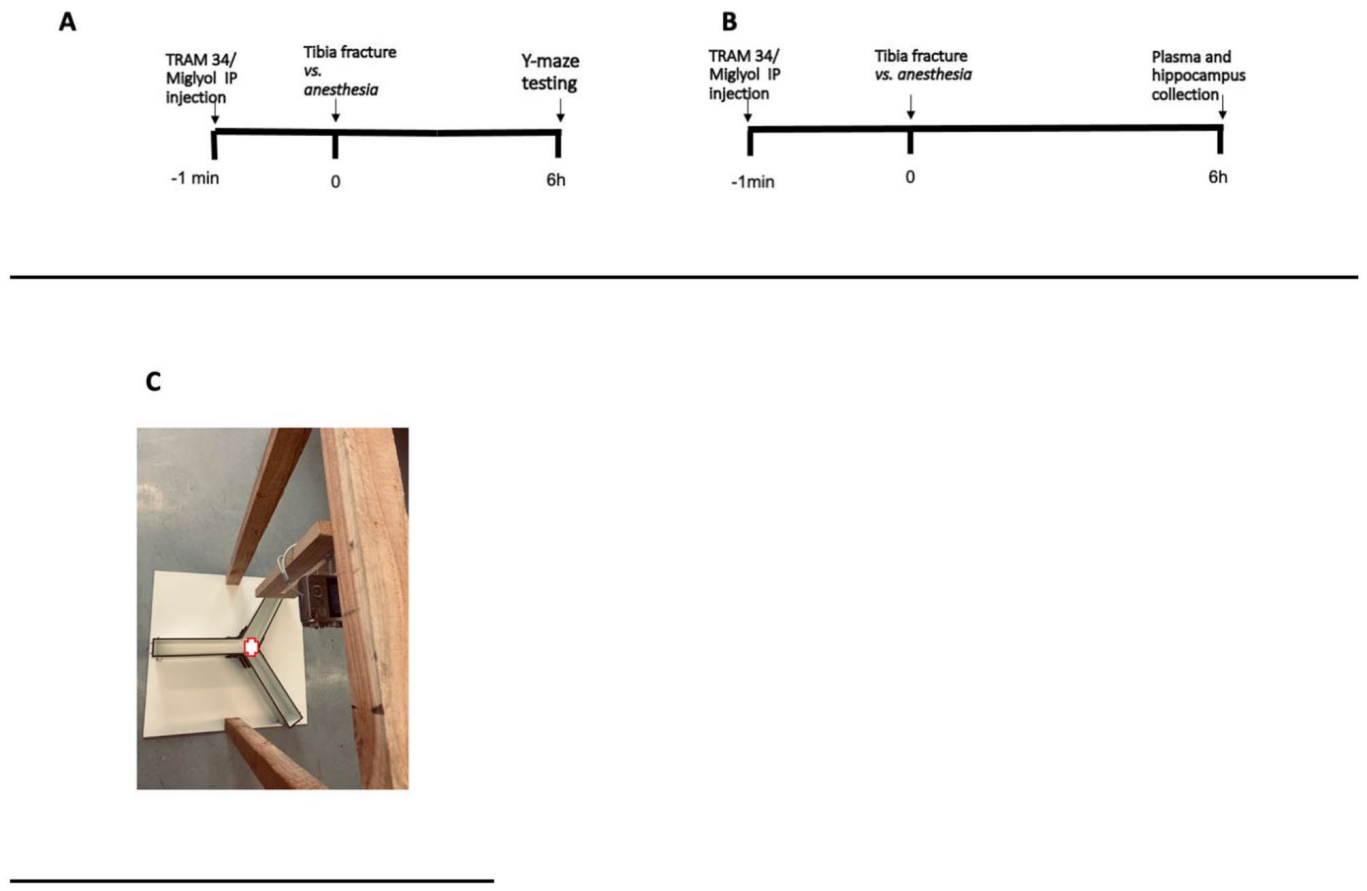
Timeline of experiments performed in *WT* mice. **A**. Timeline of the experiments to measure cognitive outcome. **B**. Timeline of the experiments to measure peripheral and hippocampal parameters. Tibia fracture was applied after anesthesia to the surgery groups. **C**. Illustration of the 3-armed Y-maze device. Each arm had the following dimensions: 35 × 6 cm; wall height: 15 cm. Angles between arms were 120 °. Each mouse was placed in the middle of the Y-maze (as indicated on the image)

Experiments were conducted in the same animal surgery room in the morning. (Fig. 1)

### Anesthesia

Mice randomized to ‘anesthesia groups’ were subjected to inhalation anesthesia (3% isoflurane in 30% FiO_2_) as well as subcutaneous buprenorphine (0.1 mg/kg).

### Surgical procedure

Under general anesthesia with 3% isoflurane in 30% FiO_2_, rodents underwent an open tibial fracture of the left hind paw with intramedullary fixation under aseptic conditions. Briefly, buprenorphine (0.1 mg/kg) was injected subcutaneously to provide analgesia after the induction of anesthesia and before skin incision. A 20 G pin was then inserted in the intramedullary canal of the left tibia, and osteotomy was performed after the periosteum was stripped. During the surgical procedure that lasted approximately 10 min, temperature was monitored and maintained at 37 °C with the aid of warming pads [8, 13].

### Intra-peritoneal injections

TRAM34 and its vehicle miglyol were provided by the Department of Pharmacology, University of California Davis, Davis, CA, USA (Prof. Heike Wulff).

TRAM34 was synthesized as previously described [19] and was dissolved at a concentration of 8 mg/ml in miglyol 812 neutral oil (Neobee M5®; Spectrum Chemicals), a low viscosity vehicle that is used as a pharmaceutical excipient and well tolerated following i.p., s.c. or oral administration.

TRAM34, 40 mg/kg or miglyol alone were injected intraperitoneally approximately 1 min before surgery.

Other reagents are detailed in the respective methodological subsections.

### Assessment of cognitive score by the Y-maze test

“Y-maze test evaluates the mice’s willingness to explore new environments. Rodents typically prefer to investigate a new arm of the maze rather than returning to one that was previously visited. Many parts of the brain, including the hippocampus are involved in this spatial memory test [22].

Mice were not trained before experiments. Six hours after surgery, mice were individually placed in the middle of a Y designed maze (each arm designated A/B/C) (Fig. 1C) and their movement was recorded for 5 min with a camera (Sony DSC-HX50). After 5 min, mice were taken out of the Y-maze and sacrificed. The Y maze was devoid of food and was thoroughly cleaned with ethanol after each recording. The following parameters from the recording were manually obtained and blindly analyzed by an independent researcher: total distance traveled in the maze, total number of arm entries, number of entries made into each arm, and the sequential list of arms entered to assess number of alternations made.

In this study, cognitive score refers to the number of alternations in the Y-maze test.

A significant decrease in alternations is a sign of a cognitive deficit [22].”

### Hippocampal microglial presence

After the behavioral assessment was completed, mice were sacrificed and tissue samples collected as previously described [17].

To analyze microglial proliferation, hippocampus was harvested and fixed in 4% formaldehyde (Klinpath, Duiven, Netherlands) and embedded in paraffin. Three µm-thick biopsy slices were cut with a microtome Leica RM2255 (Wetzlar, Germany) and mounted on microscope slides. Deparaffinised tissue sections were pretreated with hot citrate buffer pH6 and endoperoxydases were inactivated by a methanol/H_2_O_2_ treatment. After blocking (ScyTek Laboratories, UT, USA), hippocampal sections were incubated for 30 min at room temperature with a specific primary antibody (Anti-Iba1 antibody [EPR16588], Abcam, Cambridge, UK) at a 1:500 dilution. Signal amplification was performed by incubation with the complex UltrasenseStreptavidinePeroxydase RTU (ScyTek Laboratories, UT, USA). Revelation was performed with diaminobenzidine (DAB) (Biogenex, CA, USA). Slides were counterstained with hematoxylin (Cell Signaling, MA, USA). Five fields of each stained slide were acquired with 20x objective. The area percentage of Iba1 staining was obtained by colour deconvolution with Image J (NIH, Md, USA).

### Systemic inflammatory response

At the time of sacrifice, blood was harvested in heparin-coated syringes from the inferior vena cava of wild-type mice under terminal isoflurane anesthesia. Plasma was collected after centrifugation of the blood at 10,000 x g for 10 min at room temperature and stored at -80 °C for later analysis. Plasma IL-6 and HMGB1 concentrations were measured using the IL-6 ELISA kit from Millipore corporation (MO, USA) and mouse HMGB-1 Elisa kit (Novus Biologicals (Biotechne, MN, USA), respectively. Plasma samples were diluted twice with the Sample Diluent Buffer of the kit. The standard curve and samples were performed in duplicates. The absorbance was read at 450 nm with an Emax Plus Microplate Reader (Molecular Devices, CA, USA). The standard curve and the samples were performed in duplicates and the absorbance was read at 450 nm.

### Statistics

Analysis for normality revealed a non-parametric distribution and the data are displayed graphically with boxplots based on the calculation of the median (P50) and the interquartile range (IQR: P25–P75). Anesthesia and surgery experimental groups were compared by the Kruskal-Wallis test. The p-value was obtained by the non-parametric Wilcoxon Rank Sum Test. The results were considered as significant below the 5% critical level (p < 0.05). Calculations were carried out by Sigma Plot version 12.0 (Systat Software, Chicago, IL).

## Results

In wild-type (WT) mice, in the absence of TRAM34 administration, surgery was associated with a decrease in cognitive score (alternations in Y-maze), suggesting the presence of cognitive decline 6 h post-operatively (P = 0.019).

**Fig. 2:**
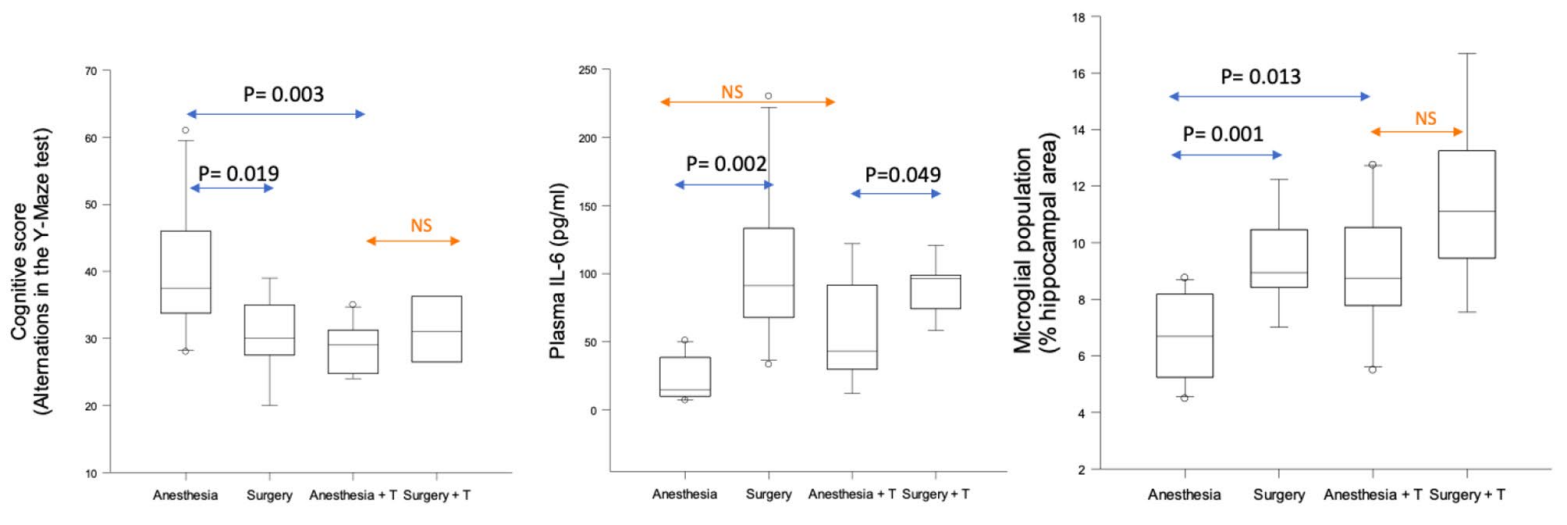
Anesthesia/ surgery-dependent changes in cognitive decline, peripheral inflammation and central proliferation of microglia in wild type mice (10 animals in each experimental group) treated with miglyol or miglyol + TRAM34 (T). Experimental groups included 10 animals. NS: not significant; T: TRAM34.

**Fig. 3:**
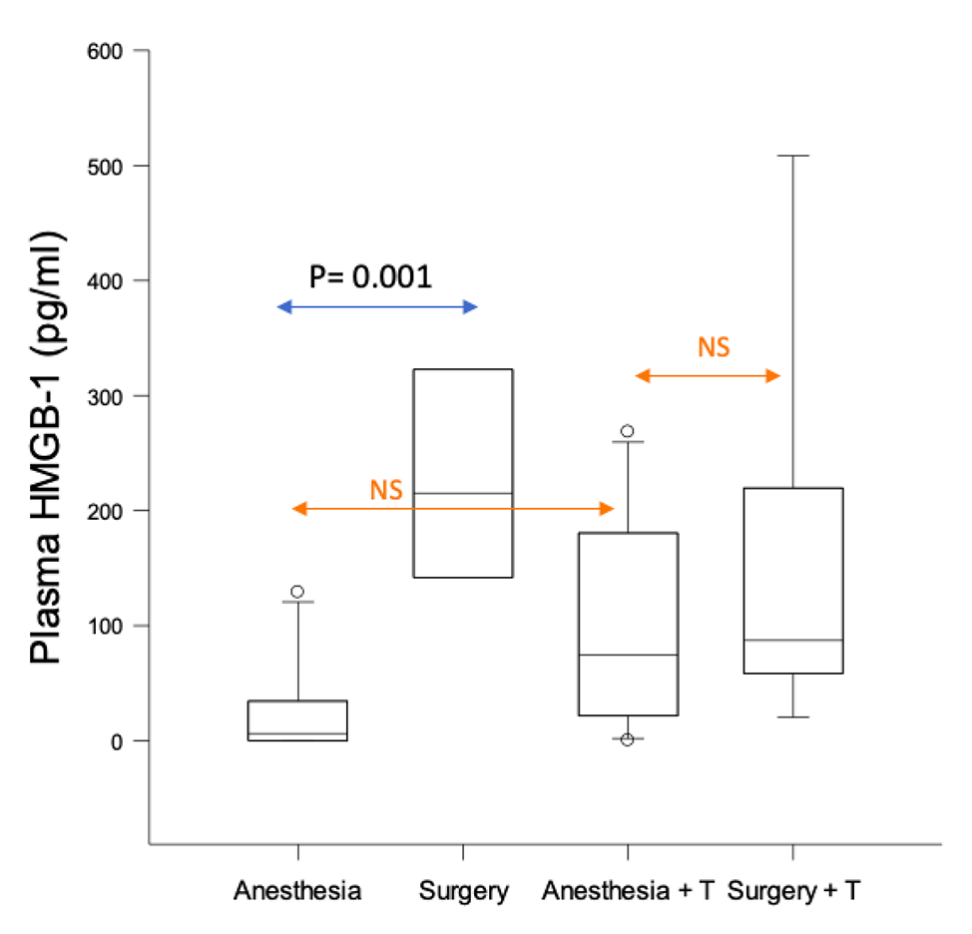
Modulation of surgery-induced rise in peripheral HMGB1 by TRAM34. Comparison of post-surgical changes (surgery vs. anesthesia) in peripheral HMGB1 levels between miglyol (reference conditions) and miglyol + TRAM34 (T) (challenge conditions) in WT mice. Experimental groups included 10 animals. NS: non-significant

In parallel, surgery also induced an increase in plasma IL-6 (P = 0.002), HMGB1 (P = 0.001), and microgliosis (P = 0.001) when compared to anesthesia alone (Fig. 2 and S1; Fig. 3).

Taken together, these results confirm the surgery-induced deleterious cognitive and neuroinflammatory effects observed in previous experimental settings [13, 17].

Based on these results, we then analyzed whether pharmacological inhibition of KCa3.1 channel by TRAM34 would influence the surgery-induced effects. As shown in Fig. 2 and S2, neither cognitive decline nor microglial proliferation was induced by surgery in the presence of TRAM34. Surgery was associated with an increase in peripheral IL-6, regardless of TRAM34 administration (P = 0.049). Surgery-induced peripheral HMGB1 increase was attenuated by TRAM34 (Fig. 3).

Parenthetically, comparison of anesthesia groups (anesthesia + vehicle vs. anesthesia + TRAM34) revealed a decrease in spatial memory cognitive score (P = 0.003) and an increase in microglial proliferation (P = 0.013) (Fig. 2).

**Fig. 4:**
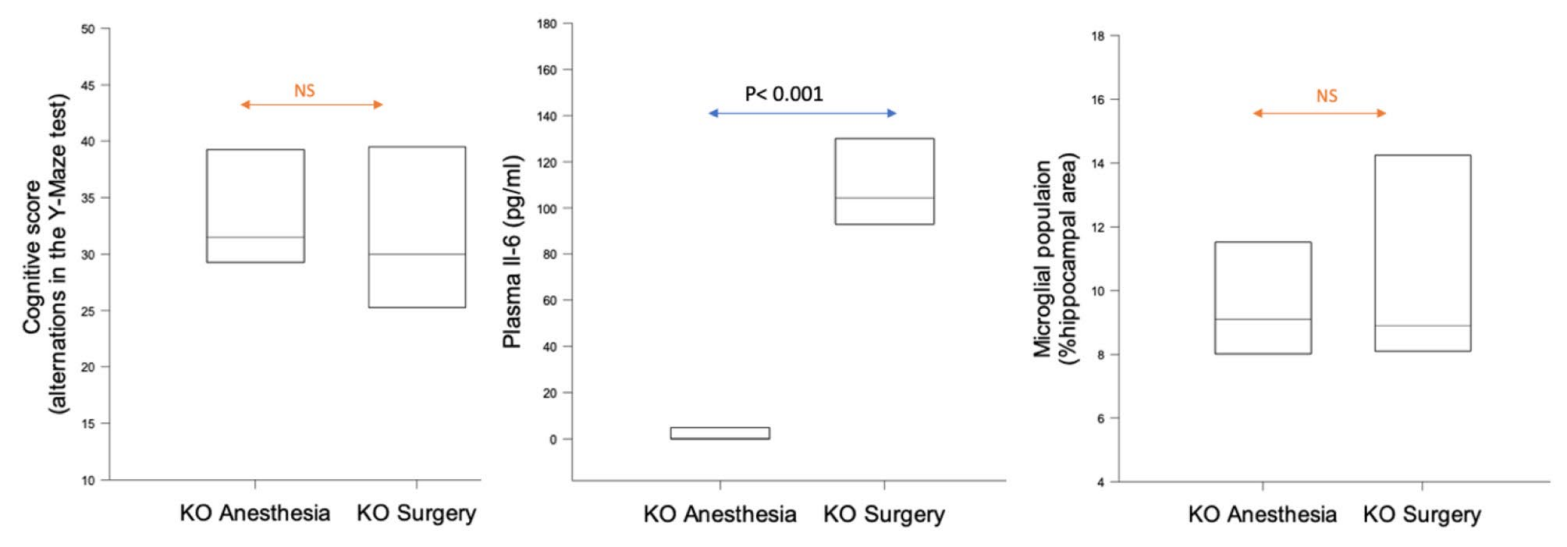
Surgery-dependent changes in cognitive decline, peripheral inflammation and central microglial presence in *KCa3.1*^*−/−*^ KO mice. Experimental groups included 10 animals. NS: non-significant

In *KCa3.1*^*−/−*^ KO mice, surgery neither induced cognitive decline nor microglial proliferation (Fig. 4) although plasma IL-6 was increased (p < 0.001).

## Discussion

### Summary of findings

The ‘surgical phenotype,’ comprising of memory impairment, microgliosis, (proliferation and amoeboid appearance), peripheral inflammation (IL-6) and upregulation of the trauma marker (HMGB1) was present in *WT* mice after aseptic trauma in the presence of miglyol only (Figs. 2 and 3). Blocking KCa3.1 channel pharmacologically (through TRAM34 administration) (Figs. 2 and 3) or genetically (through *KCa3.1* gene inactivation in the *KCa3.1*^*−/−*^ mice) (Fig. 4) mitigated the surgical phenotype (apart from circulating IL-6; see below), highlighting a previously unreported potential role of blockade of the KCa3.1 channel for prevention of PND [18].

### KCa3.1 blockade and peripheral inflammation

Postoperative IL-6 increase persisted after TRAM34 administration in *WT* mice (p = 0.049) as well as in *KCa3.1*^*−/−*^ mice; similarly, blockade of Kv1.3 channels by PAP-1 did not affect postoperative elevation of circulating IL-6 levels [16]. IL-6 is a potent propagator of the innate immune response to trauma [11] which can both transform into neuroinflammation and facilitate wound healing depending on the involved signaling mechanism [23–25]. Interestingly, the umitigated rise in postoperative IL-6 in the presence of PAP-1 was associated with no deterioration in wound healing suggesting that this peripheral cytokine is regenerative, likely through classical signaling [26]. The influence of TRAM34 adminstration on wound healing remains to be investigated.

### KCa3.1 blocking and HMGB1 release

Previous studies supported a putative causal role of surgical-trauma induced HMGB1 in PNDs [8, 27]; TRAM34 administration decreased postoperative elevation in peripheral HMGB1 levels.

As neutralizing HMGB1 prevented the development of PND [8], peripheral HMGB1 levels by themselves might play a more important role in PND development than previously considered.

### KCa3.1 blocking and cognition

Previous research indicated the role of TRAM34 in protecting memory performance in murine Alzheimer’s disease [28–30]. TRAM34 also reduced infarction and improved neurological scoring in murine stroke models [21].

Similarly, our data illustrate that KCa3.1 blocking mitigated immediate PND development.

## Limitations

### Inflammatory markers

While only two markers of inflammation and trauma (IL-6; HMGB1) were consistently measured in these experiments, other markers of inflammation also play a key role in PND development and should be studied [11, 13]. Previous research has, however, shown the importance of these markers in PND development [8, 12].

### TRAM34 administration in anesthesia control groups

The KCa3.1 inhibitor TRAM34 had a negative effect on cognition and microgliosis in the anesthesia alone *WT* mice. (Fig. 3); this interesting finding requires further exploration of the possible interaction between TRAM34 and volatile anesthetic agents.

### Blank control group

In this study, ‘anesthesia + vehicle’ was considered to be the blank/ baseline reference for the intervention e.g. Surgery/ TRAM34 injection. An ‘entirely’ blank/ baseline group was not included in the experiments.

### Which Kca3.1 channel blocking property is paramount?

As previously mentioned, KCa3.1 channels are not only present on microglia, but also on cell types such as astrocytes, macrophages, T cells and erythrocytes [16].

In this study, experiments show that TRAM34 administration decreased post-operative microgliosis. The effect of TRAM34 administration on astrogliosis was, however, not studied.

TRAM34 administration also had a peripheral effect: decreasing post-operative plasma HMGB1 levels. As monocytes play an important role in the inflammatory cascade leading to PND development [8], TRAM34 administration may also block KCa3.1 channels present in these cells.

### Kv1.3 and KCa3.1 channels

As this report and previously published data [21] have established the roles of KCa3.1 and Kv1.3 in PND development, simultaneous administration of TRAM34 and PAP-1 should be investigated in order to determine the specific role of each channel and whether an interaction, including synergism, might take place between these channel blockers.

## Conclusion

Previously published data established the role of microglial Kv1.3 in perioperative neurocognitive disorders. The data presented here indicate that perioperative KCa3.1 blockade decreases immediate perioperative cognitive changes, microglial proliferation as well as the peripheral trauma marker HMGB1. It did not influence surgery-induced IL-6 production.

Future research should investigate the specific role of each channel and whether a synergistic interaction occurs between the channel blockers.

## Electronic supplementary material

Below is the link to the electronic supplementary material.

Supplementary Material 1

Supplementary Material 2

## Data Availability

The datasets used and/or analyzed during the current study are available from the corresponding author on reasonable request.

## Abbreviations

PND: Perioperative neurocognitive disorders
HMGB1: High mobility group box 1 protein
BM-DMs: Bone marrow-derived monocytes
CNS: Central nervous system
