# Supplementary material for: Involvement of KCa3.1 channel activity in immediate perioperative cognitive and neuroinflammatory outcomes
Source: BMC Anesthesiol. 2023 Mar 16;23:80. doi: 10.1186/s12871-023-02030-2 (PMC10018926; doi:10.1186/s12871-023-02030-2)
Supplement: Supplementary file 1 — Supplementary Material 1 [file 12871_2023_2030_MOESM1_ESM.docx]

**Involvement of KCa3.1 channel activity in immediate perioperative cognitive and neuroinflammatory outcomes: supplemental figures**

*Figure S1* *Representative image of microglia detection by Iba1 staining*

**
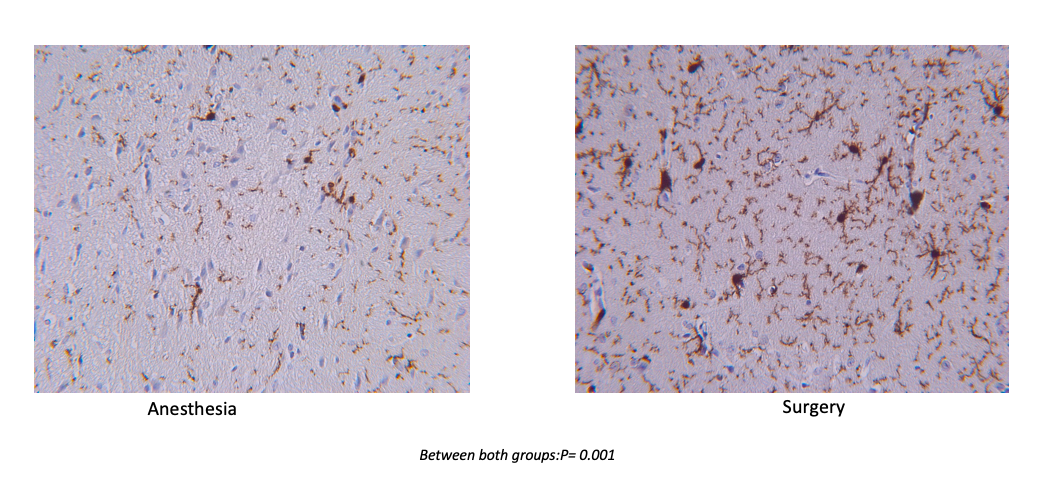
**

**Figure S1**: Representative image of microglia detection by Iba1 staining of anesthesia and surgery groups treated with miglyol (obtained by colour deconvolution with Image J (NIH, Md, USA)).

Figure S2 *Representative image of microglia detection by Iba1 staining*


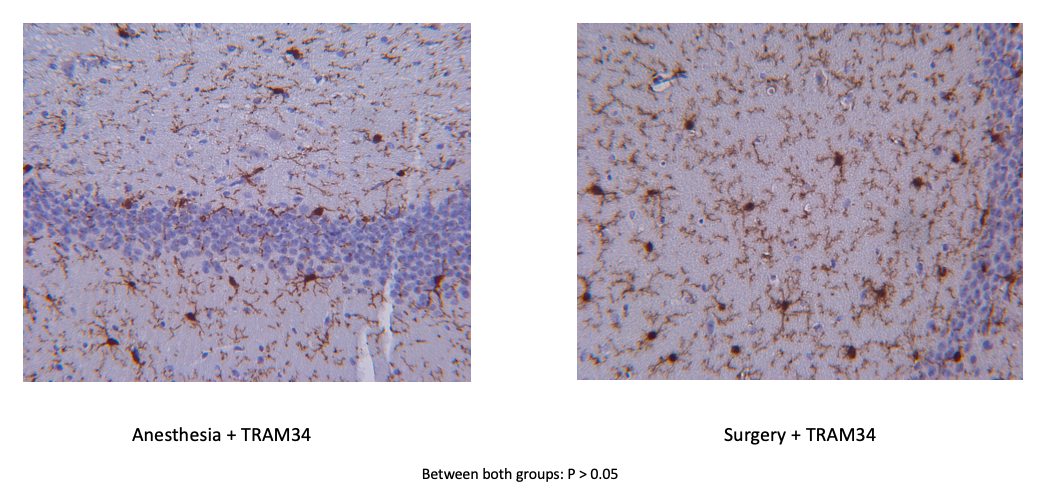


**Figure S2.**  Representative image of microglia detection by Iba1 staining of anesthesia and surgery groups treated with miglyol + TRAM-34 (obtained by colour deconvolution with Image J (NIH, Md, USA)).
